# Single-centre experience of refractory rheumatoid arthritis

**DOI:** 10.1093/rap/rkac057

**Published:** 2022-08-01

**Authors:** John Fitton, Andrew Melville, Kamran Naraghi, Jacqueline Nam, Shouvik Dass, Paul Emery, Maya H Buch

**Affiliations:** Leeds Institute of Rheumatic and Musculoskeletal Medicine, University of Leeds, Chapel Allerton Hospital; NIHR Leeds Biomedical Research Centre; Leeds Institute of Rheumatic and Musculoskeletal Medicine, University of Leeds, Chapel Allerton Hospital; NIHR Leeds Biomedical Research Centre; Leeds Institute of Rheumatic and Musculoskeletal Medicine, University of Leeds, Chapel Allerton Hospital; NIHR Leeds Biomedical Research Centre; Leeds Institute of Rheumatic and Musculoskeletal Medicine, University of Leeds, Chapel Allerton Hospital; NIHR Leeds Biomedical Research Centre; Department of Rheumatology, Leeds Teaching Hospitals NHS Trust, Leeds; Leeds Institute of Rheumatic and Musculoskeletal Medicine, University of Leeds, Chapel Allerton Hospital; NIHR Leeds Biomedical Research Centre; Leeds Institute of Rheumatic and Musculoskeletal Medicine, University of Leeds, Chapel Allerton Hospital; Centre for Musculoskeletal Research, School of Biological Sciences, University of Manchester; NIHR Manchester Biomedical Research Centre, Manchester University NHS Foundation Trust, Manchester, UK

**Keywords:** RA, refractory disease, targeted therapy, biological DMARD

## Abstract

**Objectives:**

The aim was to evaluate the proportion of RA patients who are refractory to multiple targeted therapies (TTs) in a real-world cohort of patients in a tertiary rheumatology referral centre, to describe patterns of drug sequencing associated with the development of refractory RA (RefRA) and to identify whether there is a subgroup of RefRA patients in whom successive drugs have shown primary lack of efficacy.

**Methods:**

Patients at a single centre were defined as refractory if they had failed two or more classes of TT and were identified from a dedicated TT clinic database. Reasons for drug failure were recorded, and patients were categorized pragmatically as having mild [failure of two biologic DMARD (bDMARD) classes], moderate [failure of at least three bDMARD classes] or severe [failure of at least two bDMARD classes and JAK inhibitor] refractory disease.

**Results:**

One hundred and seventy-two patients were identified as RefRA (>10% of our TT-exposed cohort); median [interquartile range (IQR)] TT exposures of four (two), 81.5% female, 82% seropositive, mean (s.d.) age of 63 (12.3) years. Detailed analysis of 60 patients showed a median (IQR) disease duration of 22 (10.75) years, median (IQR) time from diagnosis to initiation of first TT of 5 (10) years, and mean (s.d.) baseline DAS28CRP before starting first-line TT of 5.91 (0.84). Among RefRA patients, 15% were severely refractory, and 6% had demonstrated no clinical response to any TT.

**Conclusion:**

A small proportion of patients have true RefRA. Most patients fail multiple therapies owing to a combination of inefficacy and adverse events.

Key messages

- More than 10% of our single-centre targeted therapy-exposed cohort demonstrated multi-targeted therapy-refractory RA.
- The majority comprised mixed non-response, loss of response and drug toxicity.
- A rare subgroup of ∼6% of refractory RA demonstrated lack of efficacy to multiple therapies.

## Introduction

The use of biologic DMARDs (bDMARDs) and targeted synthetic DMARDs has revolutionized the treatment of RA, leading to a significant improvement in long-term outcomes. Their use, in conjunction with modern treat-to-target strategies, means that disease remission, or at least low disease activity, are realistic goals [1].

Over the last two decades, an ever-increasing number of targeted therapies (TTs) have been licensed for use in the treatment of RA. These comprise anti-cytokine molecules that target TNF-α and IL-6, molecules targeted against B cells (rituximab) and against T-cell co-receptors (abatacept). More recently, the Janus kinase inhibitors (JAKi), small molecule inhibitors of intracellular signalling molecules downstream of the receptors of multiple inflammatory cytokines, have been introduced and widely prescribed. JAKi potentially offer an important therapeutic option in advanced RA. The ability to inhibit multiple inflammatory cytokines in tandem might help to avoid the issue of cytokine redundancy, whereby multiple cytokines can perform the same biological function, which might be a reason for lack of efficacy with single cytokine inhibitors. Evidence is beginning to emerge of the efficacy of JAKi in refractory RA (RefRA) [2].

Despite this treatment revolution, 30–40% of RA patients fail to respond to their first bDMARD, and response diminishes with increasing bDMARD exposures [3]. Only 9% of patients remain on a single bDMARD for 10 years [4]. Extrapolation from randomized controlled trial evidence suggests that almost 20% of patients progress to a third bDMARD [5]. A cohort of patients who have failed multiple TTs is now evident. RefRA in the era of targeted therapies has thus emerged as a clinically challenging area and is recognized as an important area of research [6].

The definition of RefRA is complex, however. Patients fail a TT owing to lack of efficacy (typically described as primary non-response if from the outset or secondary non-response if initial response is lost) or adverse effects of drugs. Barriers to successful optimization of treatment that influence these outcomes also exist, including a lack of adherence or the effects of physical or psychological co-morbidity, further complicating RA management [7]. The concept of difficult to treat RA encapsulates this complexity but also captures those intrinsic or true refractory cases, where inflammation persists despite optimization of therapy [8].

A number of published reports describing RefRA cohorts have used different definitions of varying stringency, including failure of conventional synthetic DMARD and one TT [9], failure of two or more TTs [10], and failure of at least one anti-cytokine and one anti-cell therapy [5]. This presents challenges in determining the extent of refractory disease in the RA population. TheBritish Society for Rheumatology Biologics Register-RA study (BSRBR-RA) reported that 6% of bDMARD patients fulfilled RefRA criteria (owing to lack of efficacy and/or toxicity with at least three bDMARDs) in patients whose first bDMARD was a TNF inhibitor (TNFi) [10], whereas other authors have estimated the problem to be as high as 10–20% [6, 7]. Recent EULAR guidance defines difficult-to-treat RA as a failure of two or more TTs of differing mechanisms of action, the presence of persistent active disease, and management of RA perceived to be problematic by the physician and/or patient [11], introducing a consistency for future studies.

We aimed to determine the extent and basis of RefRA in a large, single-centre cohort of RA patients exposed to TT over the last 20 years and to describe the patterns of drug sequencing in terms of combination of loss of response, non-response or adverse effects that are associated with development of RefRA. We also aimed to identify a subgroup of RefRA patients with lack of efficacy to all available classes of TT, a group we have defined as true severe refractory RA, and to determine what proportion of the total they represent.

## Methods

### Study design

Leeds Teaching Hospitals NHS Trust (LTHT) has a cohort of >1500 patients with RA who have been exposed to one or more TT. For each patient, data on the number of TTs, sequential order of TTs, and (where available) the reason for individual drug failure have been recorded prospectively in a database and clinical records.

**Fig. 1:**
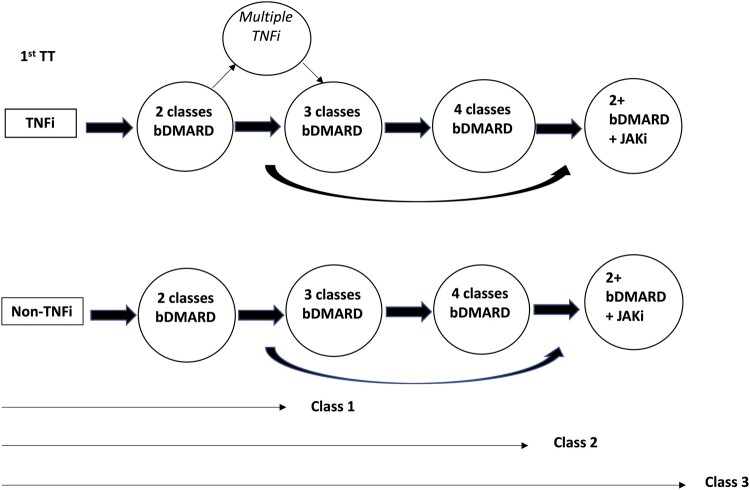
Sequence of targeted therapies in a refractory RA cohort and further pragmatic classification into three severity classes bDMARD: biologic DMARD: JAKi: Janus kinase inhibitor; TNFi: TNF inhibitor; TT: targeted therapy.

Refractory RA patients were classified as those with failure of at least two classes of TT (bDMARD and/or targeted synthetic DMARD). All patients had also failed two conventional synthetic DMARDs, as per UK commissioning guidelines on the prescription of targeted therapies, and thus this cohort also conforms with the EULAR definition of difficult-to-treat RA. The reason for drug failure was classified into one of three categories: primary non-response, where no significant clinical response was seen within 6 months of initiation of therapy; secondary non-response, where a clinical response was seen, but subsequently lost; or toxicity. Loss of response was determined by the treating rheumatologist. On a pragmatic level, the state of RefRA is reached when all potentially useful available therapeutic options have been exhausted, and this has evolved over time [8]. We used these concepts to stratify our cohort into three subclasses; class 1, who have failed two classes of bDMARDs and are on the milder end of the spectrum of refractory disease; class 2, who have failed three or four classes of bDMARDs and are thus more refractory; and class 3, who have failed at least two bDMARDs and a JAKi. This last group represents patients in whom neither bDMARDs nor the broader spectrum of cytokine blockade by JAKi has been successful. Fig. 1 shows the treatment progession of these different classes diagrammatically for patients starting both TNFi and non-TNFi first as first-line therapies.

### Patients

Patients who had failed at least two classes of TTs (i.e. exposed to three or more TT classes) were identified as they attended the clinic over an 18-month period, with further data obtained from the databases detailed above. Where needed, clinical records were obtained to confirm disease duration and DAS-28 joint count (DAS28) scores before starting TT. In some cases, long-term records could not be obtained, and the process was halted by the coronavirus disease 2019 pandemic. All patients had signed wriitten consent to the rheumatoid arthritis disease research study (RADAR). Ethical approval for this study was granted by Leeds West Research ethics committee.

### Statistical analysis

Patient demographics and clinical characteristics were summarized for each group using proportions of patients, median with interquartile range (IQR) or mean with s.d. as appropriate, and percentages of mild, moderate and severe RefRA were calculated.

## Results

One hundred and seventy-two patients were identified as having failed two or more TTs [median (IQR) four (two)]; 81.3% were female, 80.1% seropositive, with a mean (s.d.) age of 63 (12.3) years. Disease duration and baseline DAS28CRP results were available for a sample of 60 patients, with results as follows: median (IQR) disease duration was 22 (10.75) years, median (IQR) time from diagnosis to initiation of first TT was 5 (10) years, and mean (s.d.) baseline DAS28CRP before starting a first-line TT was 5.91 (0.84).

Reasons for drug discontinuation were identified in 166 cases. One hundred and fifty-two of these patients had failed both an anti-cytokine and a cell-targeted therapy. Table 1 shows the numbers in each category of RefRA.

**Table 1:** Demographics, first-line targeted therapy and reasons for drug switching in whole cohort and by classification of severity of refractory disease

	Whole group (*n* = 166)	Refractory RA category		
		Class 1 (two classes of bDMARD)	Class 2 (three/all four classes of bDMARD)	Class 3 [multi-bDMARD (minimum of two classes) + JAKi]
Number of patients	166	64^a^	76	26
Age, mean (s.d.), years	63 (12.3)	62 (12.5)	64 (11.6)	61 (14.3)
Female, *n* (%)	135 (81.3)	51 (79.7)	64 (84.2)	20 (76.9)
RF^+^ and/or anti-CCP^+^, *n* (%)	134 (80.1)	48 (75)	66 (86.8)	20 (76.9)
First targeted therapy, *n* (%)				
TNFi	139 (83.7)	54 (84.4)	61 (80.3)	24 (92.3)
Rituximab	15 (9)	5 (7.8)	9 (11.8)	1 (3.8)
Tocilizumab	4 (2.4)	3 (4.7)	1 (1.3)	0 (0)
Abatacept	8 (4.8)	2 (3.1)	5 (6.6)	1 (3.8)
Reason for drug discontinuation, *n* (%)				
Mixed NR (primary and/or secondary)	65 (39.2)	31 (48.4)	28 (36.8)	6 (23.1)
Primary NR only	10 (6)	5 (7.8)	3 (3.9)	2 (7.7)
Secondary NR only	13 (7.8)	7 (10.9)	6 (7.9)	0 (0)
Mixed NR and toxicity	72 (43.4)	19 (29.7)	36 (47.4)	17 (65.4)
Multiple toxicity	6 (3.6)	2 (3.1)	3 (3.9)	1 (3.8)

a Forty-nine patients received one anti-cytokine and one cell-targeted bDMARD, 12 received two anti-cytokine bDMARDs (TNFi and tocilizumab), and 3 received two cell-targeted treatments (rituximab and abatacept).

anti-CCP: ant-CCP antibody; bDMARD: biologic DMARD; JAKi: Janus kinase inhibitor; NR: non-response; TNFi: TNF inhibitor.

Fifty patients had received multiple (at least two) TNFi and at least one other class of TT [80% female, 76% seropositive, mean (s.d.) age 63.5 (14.3) years], reflecting a period with more limited treatment options. Of these, 39 were switched to a cell-targeted therapy (31 rituximab and 7 abatacept), 36 of whom had maintained their response to their cell-targeted therapy. Eleven of 50 were switched from a TNFi to tocilizumab.

**Fig. 2:**
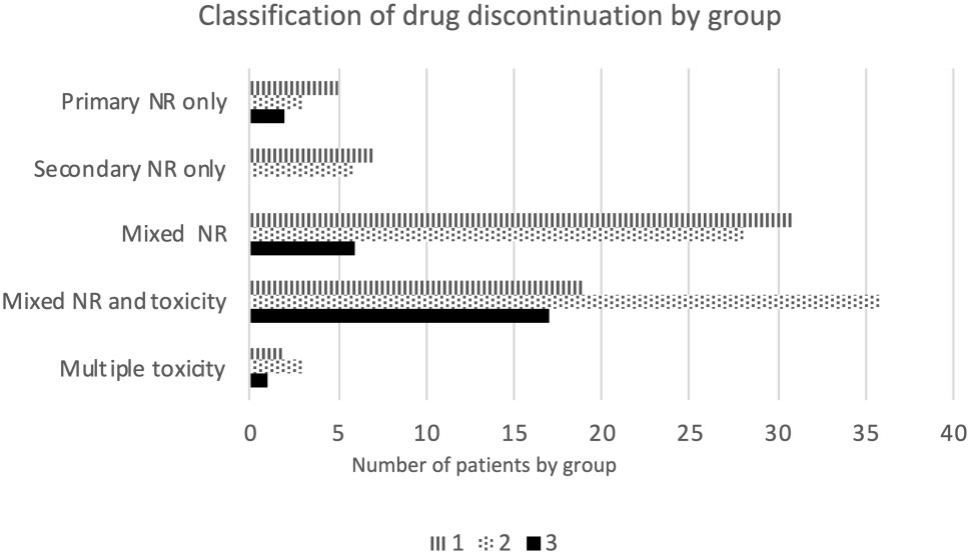
Reasons for drug discontinuation within each refractory RA severity subgroup NR: non-response.

Eighty-eight patients in total demonstrated only non-response to targeted therapies, with no history of significant adverse effects leading to a cessation of treatment. Eight of these cases had failed all four classes of bDMARDs and a JAKi, although one of these patients subsequently derived a response to a second JAKi. Sixty-five patients demonstrated a combination of primary non-response and secondary loss of response, including 28 of 76 exposed to three or more bDMARD classes and 6 of 26 patients exposed to two or more bDMARDs and a JAKi. Thirteen patients responded initially to all their treatments, but subsequently lost response (sequential secondary failure). Ten patients showed successive primary non-response to two or more bDMARD classes, and five patients to three or more classes of TT. Two had severe (class 3) refractory disease, with multiple primary drug failures. Fig. 2 shows reasons for drug discontinuation by group.

## Discussion

More than 10% of our cohort of patients treated with TT have RefRA, defined as non-response to two or more bDMARD classes, in keeping with the EULAR definition of difficult-to-treat RA. A similar proportion failed both an anti-cytokine and cell-targeted therapy. Of this cohort, 15.7% had severe refractory disease according to our pragmatic classification system that, unlike published reports to date, captures JAKi use. All of these 26 patients had tried and failed at least one anti-cytokine therapy, one anti-cell-targeted therapy and a JAKi.

Our cohort includes a larger (albeit still modest) proportion of patients with RefRA than that identified in a BSRBR report (6%) [10]. The BSRBR report included only patients whose first-line TT was a TNFi. Although our cohort included patients who started any TT first line, the majority (80%) used a TNFi, reflecting UK practice. In many cases, the choice of first-line bDMARD (and subsequent sequencing) reflected the order in which the different bDMARDs came on to the market. This is represented in the fact that 77 (44.8%) of our refractory cohort cycled through two or more TNFi before receiving a non-TNFi bDMARD. TNFi cycling is shown to be effective, even in the case of non-response to a first-line TNFi [12, 13], but on a group level, switching class of biologic is likely to be more effective [14, 15]. The lack of alternative targets for therapy in those who started bDMARDs in the early 2000s might have contributed to the refractory nature of their disease. In the BSRBR study of RefRA, patients registered more recently were likely to drug cycle more readily and to meet the definition of RefRA more quickly because of the wider array of treatments available to patients now, compared with earlier in the bDMARD era [10].

Our data show that in a large proportion of cases (>40%), patients fail multiple TTs owing to a mixture of inadequate response and adverse effects. Only six patients in total had multiple drug intolerances as their sole reason for cycling therapies. More than 50% of patients, however, failed multiple drugs owing to a lack of efficacy, highlighting the challenges in delivering precision medicine. We describe non-response as mixed primary and secondary non-response. Primary drug failure is characterized by an absence of response at the earliest time of clinical assessment (and often varies between 12 and 24 weeks), and has traditionally been hypothesized to be attributable to mismatch between drug target and disease target, whereas secondary failure acquired following an initial response might be attributable to underlying drug immunogenicity [5]. However, the value of this distinction in providing a possible biological context is not clear, because these definitions can be imprecise, and determination of response status falls within a spectrum. In addition, evidence that drug cycling within class can be efficacious even in the event of a primary non-response [12] challenges this paradigm. Interestingly, 10 patients (6% of the refractory cohort) have shown no response to any TT to date, including one patient who is in the severe category and has failed a total of eight targeted therapies. These patients represent a rare and true refractory group of the contemporary era.

The absence of predictive biomarkers of drug response and the introduction of cost-effective biosimilars has contributed to TNFi remaining the predominant first-line TT, and thus, identifying an association between sequence of therapies and refractory outcome is not feasible. In practice, a number of factors contribute to the treatment decisions, including patient characteristics, disease phenotype, physician and patient preference, and cost. The presence of co-morbidity (e.g. malignancy, lung disease, recurrent infection) is an important consideration when starting and cycling TTs. There is evidence to suggest that clinicians might favour the use of certain non-TNFi bDMARDs in patients with co-morbidity owing to their perceived favourable safety profile [16]. Multimorbidity has been shown to reduce the chances of obtaining low disease activity [17] and has been identified as a risk factor for difficult-to-treat RA in an international survey of rheumatologists [7]. We do not have the necessary data in our cohort to describe wider difficult-to-treat characteristics. However, the inclusion of patients starting non-TNFi bDMARDs in our report might reflect a more multi-morbid cohort and be another reason why our cohort appears more refractory than that evaluated by the BSRBR.

The proportion of females in our RefRA cohort (81.5%) is higher than that of a general RA population. Female sex is a recognized risk factor for a refractory disease [9]. Other risk factors include delay to starting TT and higher disease activity at baseline. Our analysis of a subset of 60 patients shows an average time from diagnosis to starting a bDMARD of 5 years, reflecting disease onset preceding the bDMARD era in a proportion of our cohort. These patients would have had exposure to a greater number of conventional synthetic DMARDs before initiation of TNFi, which is a risk factor for a worse response to therapy [18]. Early intervention and tight disease control prevent disease progression and structural damage in RA, leading to better outcomes [19]. We have recently highlighted the importance of recognizing non-inflammatory refractory RA; that is, apparent refractory patients in whom measured raised disease activity (that triggers drug cycling) is predominantly driven by pain in the absence of joint and/or systemic inflammation [8]. This aligns with the EULAR definition of difficult-to-treat RA [11] that includes individuals with well-controlled disease but the persistence of RA symptoms that are causing a reduction in quality of life. Structural damage and pain sensitization are likely to be contributors to this profile and might be relevant in a number of patients within our cohort.

The main limitations of this study are that it is based on a modestly sized historical cohort, with no control group. It is therefore not suitable for regression analysis to investigate risk factors for refractory disease, nor do we have wider outcome data to enable a detailed assessment of the consequences of refractory disease. We have presented a single-centre experience of RefRA from a tertiary referral centre. Therefore, this review must also come with the usual caveat that it might not be generalizable to other institutions with different patient populations and different approaches to management of RA with TTs. However, our result of ∼10% of patients within our cohort having RefRA is comparable to other studies in the area [10, 20, 21].

Nevertheless, our descriptive data contribute to a better understanding of the nature and extent of RefRA, and within this spectrum we identify a subgroup with multiple TT inefficacy and severe RefRA. Characterizing these cohorts systematically with experimental studies will be crucial to inform on optimal management strategies and novel targets for drug development.

*Funding:* This paper presents independent research funded/supported by the National Institute for Health Research (NIHR) Leeds Biomedical Research Centre (BRC). The views expressed are those of the author(s) and not necessarily those of the NIHR or the Department of Health and Social Care.

*Disclosure statement:* J.F. has received speaker fees from Pfizer. P.E. has received consultant fees from AbbVie, BMS, Eli Lilly, MSD, Novartis, Pfizer, Roche, Samsung, Sandoz and UCB and received research grants paid to his employer from AbbVie, BMS, Pfizer, MSD and Roche. M.H.B. has provided expert advice and received consultant fees from AbbVie, Bristol-Myers Squibb, Eli Lilly, EMD Serono, Pfizer, Roche, Sandoz, Sanofi and UCB and has received research grants paid to her employer from Pfizer, Bristol-Myers Squibb Ltd, Roche and UCB. The remaining authors have declared no conflicts of interest.

## Data Availability

Data are available upon reasonable request by any qualified researchers who engage in rigorous, independent scientific research, and will be provided following review and approval of a research proposal and Statistical Analysis Plan (SAP) and execution of a Data Sharing Agreement (DSA). All data relevant to the study are included in the article.
